# Management of posttraumatic posterior shoulder instability following a Latarjet: a case report

**DOI:** 10.1016/j.xrrt.2024.03.010

**Published:** 2024-04-10

**Authors:** Timothy Kanne, John Lusk, Nicholas Adam Howard, Brent Ponce, Bassem Elhassan

**Affiliations:** a Edward Via College of Osteopathic Medicine, Auburn, AL, USA; b Mercer School of Medicine, Savannah, GA, USA; c Hughston Clinic, Columbus, GA, USA; d Harvard Medical School, Boston, MA, USA

**Keywords:** Posterior, Shoulder, Instability, Post, Ipsilateral, Latarjet

A common complication of shoulder dislocations is recurrent instability, as the soft tissue restraint providing stability is commonly damaged.^23^ Recurrent instability is especially high in younger contact athletes and in patients with some degree of either glenoid (ie bony Bankart) or humeral (ie Hill-Sachs) bone loss.^21^^,^^22^ Surgical treatment for anterior instability commonly includes soft tissue procedures to reattach a torn anterior inferior glenohumeral ligament or Latarjet-Bristow procedures, a technique that involves transferring the patient’s coracoid process to augment a bony-deficient glenoid, and thus increase stability.^12^^,^^16^^,^^24^ Similar to anterior shoulder instability, surgical treatment for posterior instability can involve soft tissue procedures to reattach the posterior band of the inferior glenohumeral ligament or bone grafting to reconstruct or augment a bony-deficient labrum.^15^^,^^28^ The purpose of this case report is to describe the treatment and outcome of a 17-year-old male who had a posterior shoulder bony reconstruction for posttraumatic posterior shoulder instability three years after a Latarjet.

## Case report

**Figure 1:**
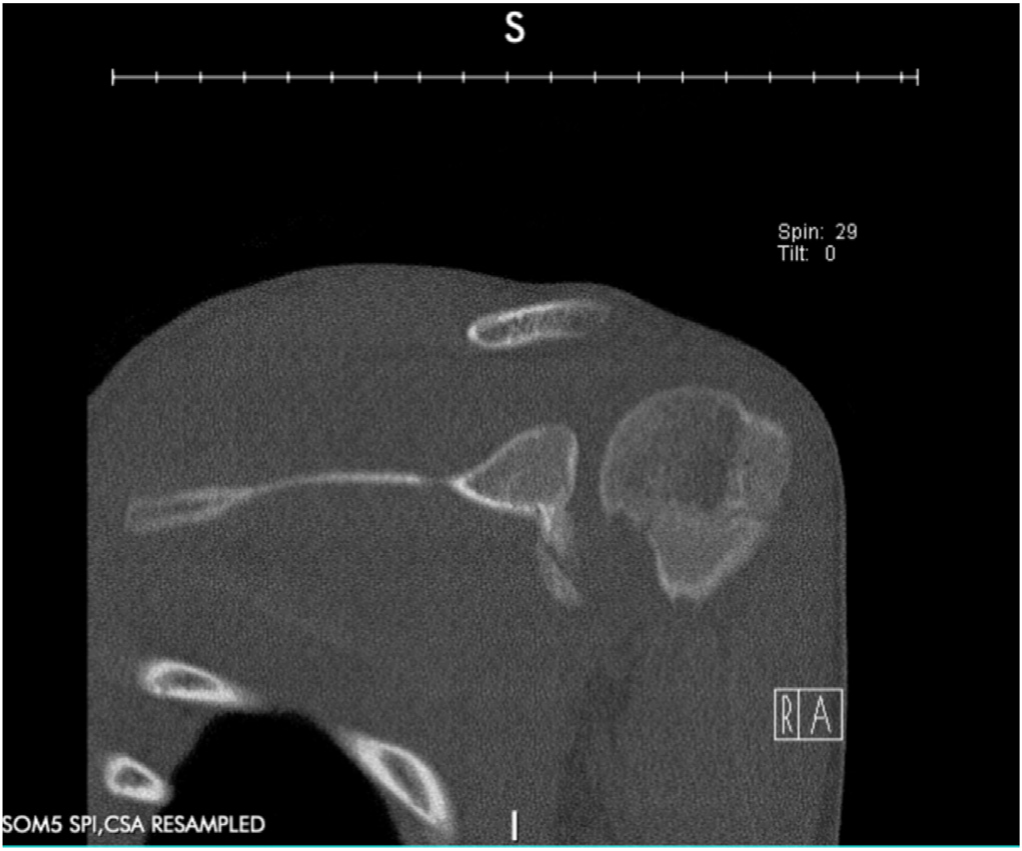
Coronal CT image taken after anterior dislocation revealing bony Bankart lesion.

**Figure 2:**
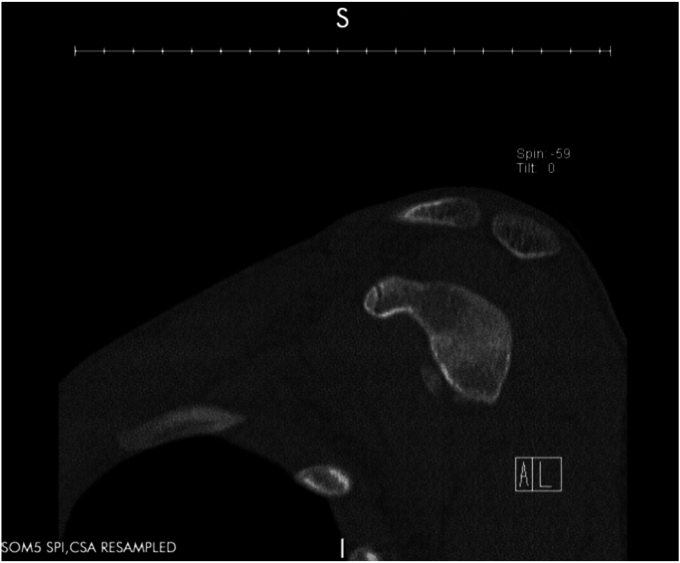
Sagittal CT image taken after anterior dislocation revealing bony Bankart lesion.

**Figure 3:**
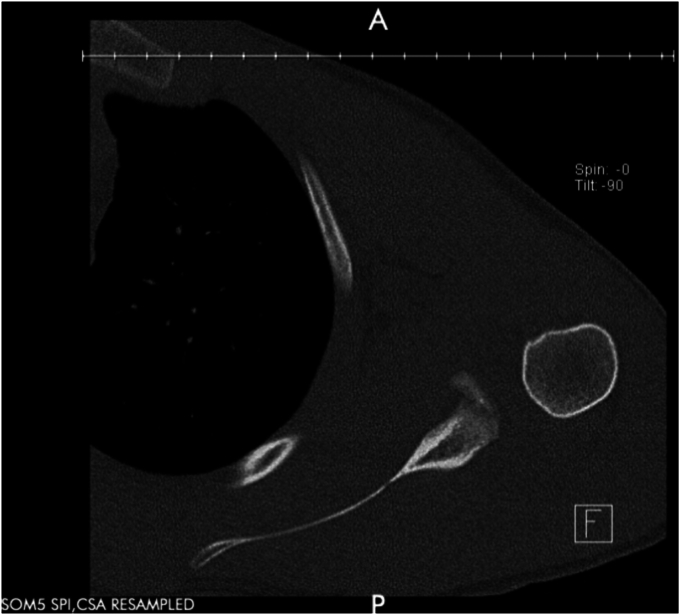
Axial CT image taken after anterior dislocation revealing bony Bankart lesion.

**Figure 4:**
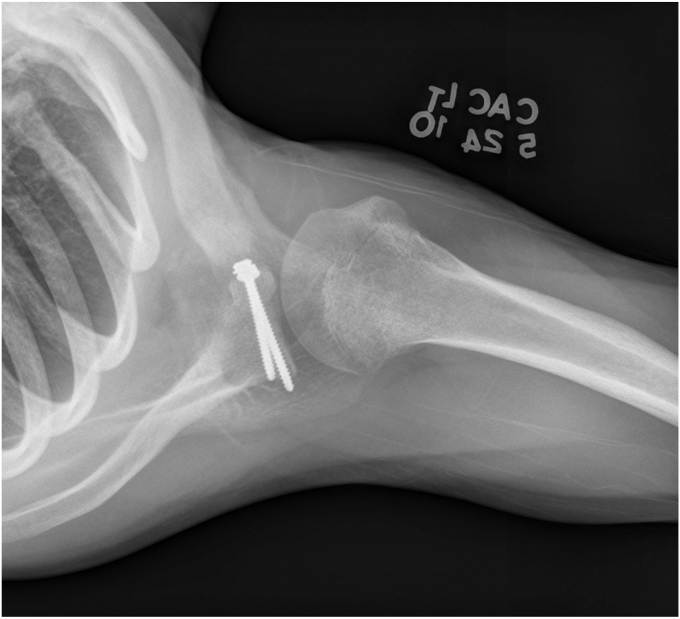
Axial X-ray from 4.5 weeks after Latarjet.

**Figure 5:**
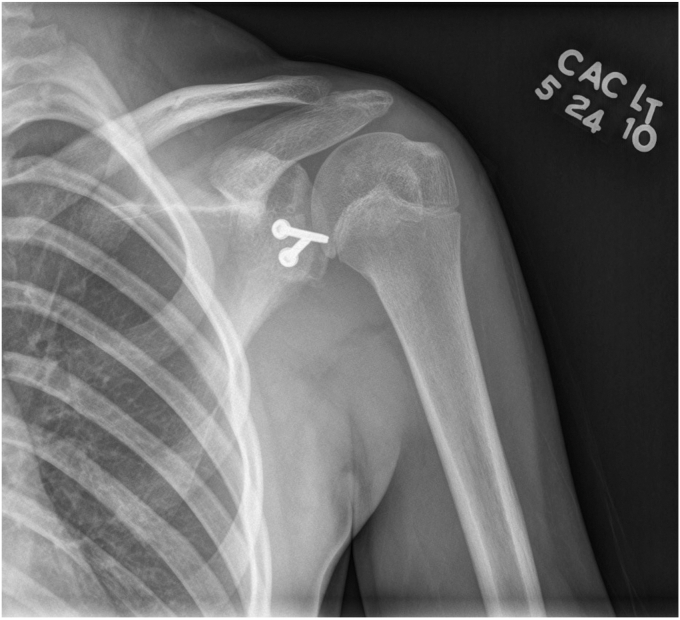
Anterior to posterior X-ray from 4.5 weeks after Latarjet.

A 17-year-old right hand dominant male presented for left posterior shoulder instability following a reported posterior dislocation with spontaneous reduction caused by an axial load injury from falling on an outstretched hand. Three years prior, he suffered an anterior left shoulder dislocation with axillary nerve palsy during competitive wrestling and subsequently underwent a closed reduction. Due to a large bony Bankart lesion (Figure 1, Figure 2, Figure 3), he underwent a primary Latarjet with neurolysis of the medial, lateral, and posterior cords of brachial plexus as well as the axillary and musculocutaneous nerve (Figs. 4 and 5). The patient regained his preinjury level of axillary nerve function and deltoid contraction with normal findings on EMG by 24 months postinjury. He lost approximately 10 degrees of internal rotation but regained full range of motion (ROM) otherwise and reached preinjury strength of the left shoulder by 24 months postinjury. He was able to return to his preinjury level of athletic participation without pain, recurrent instability, motor, and sensory deficits.

**Figure 6:**
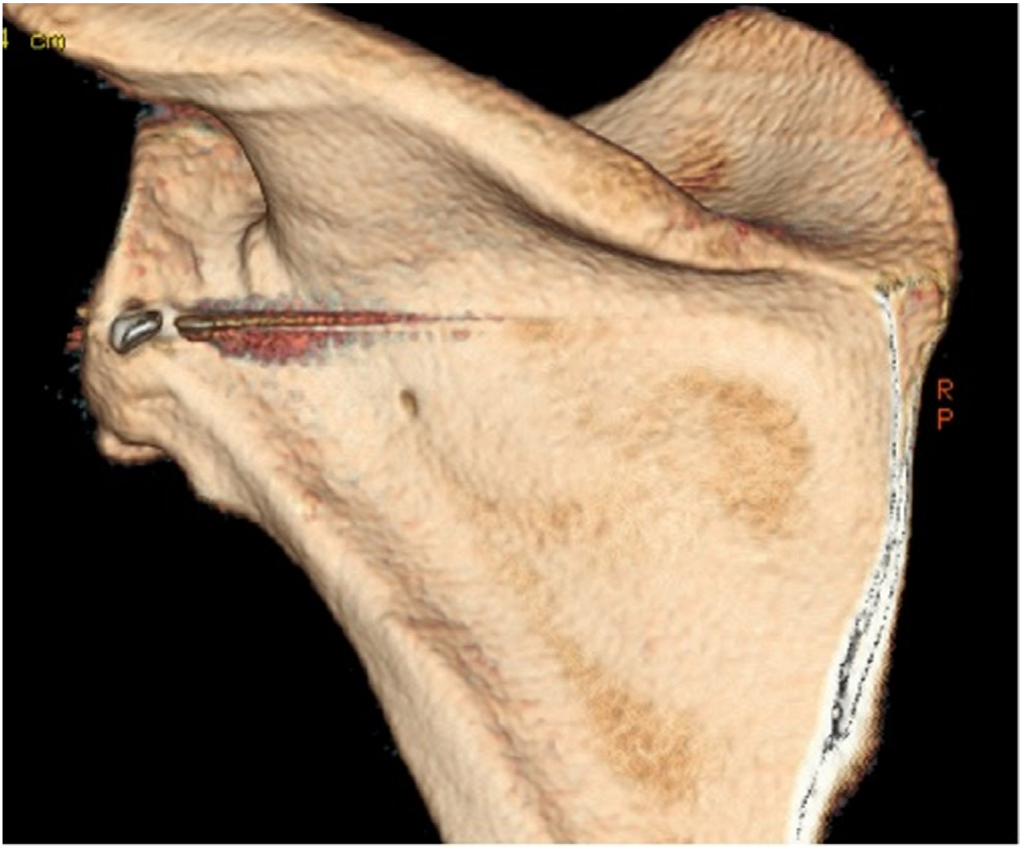
Three-dimensional CT scan image taken after posterior dislocation of posterior scapula and glenoid revealing bony deficiency of the posterior-inferior glenoid.

**Figure 7:**
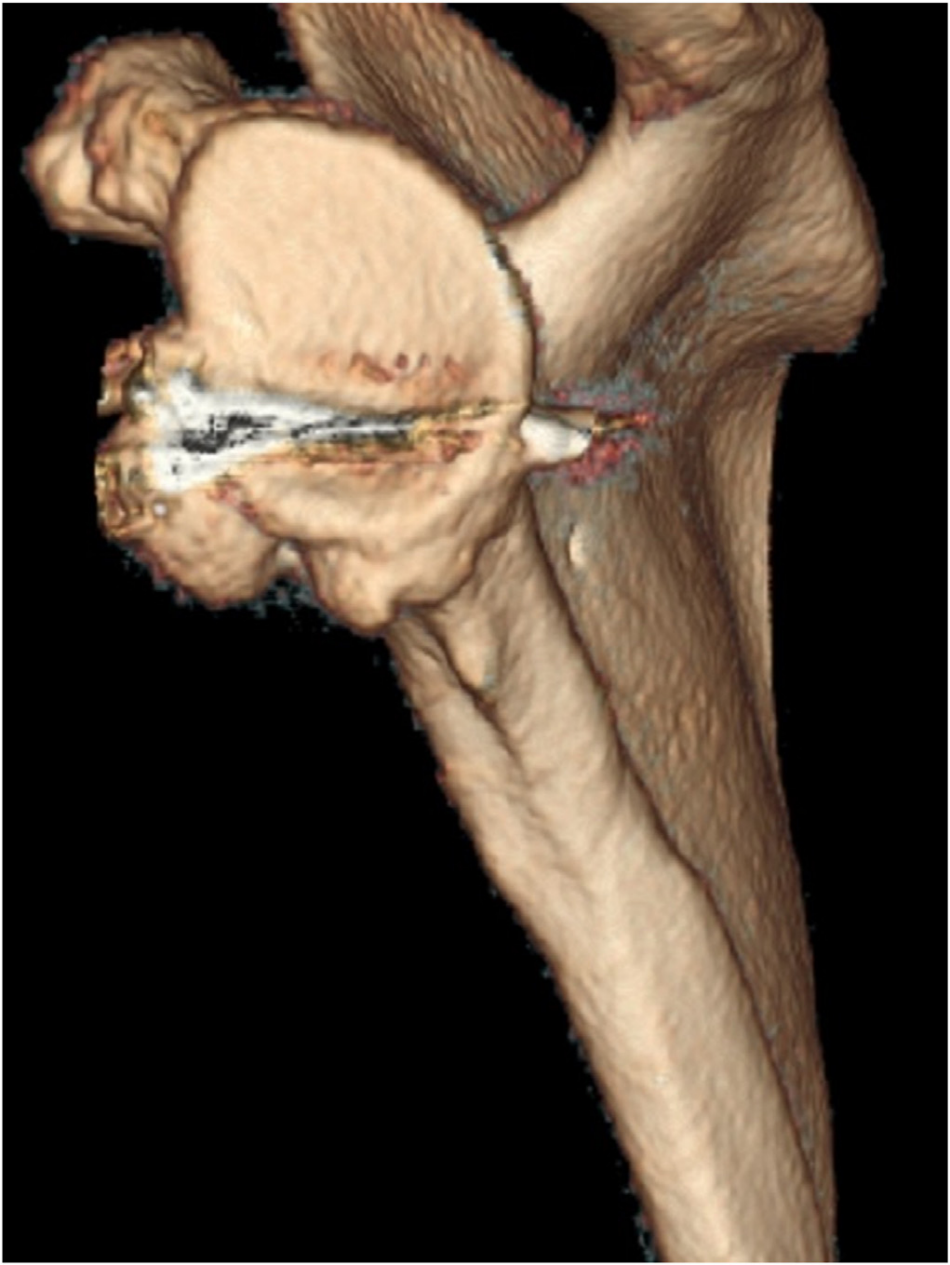
Three-dimensional CT scan image taken after posterior dislocation of lateral scapula and glenoid.

**Figure 8:**
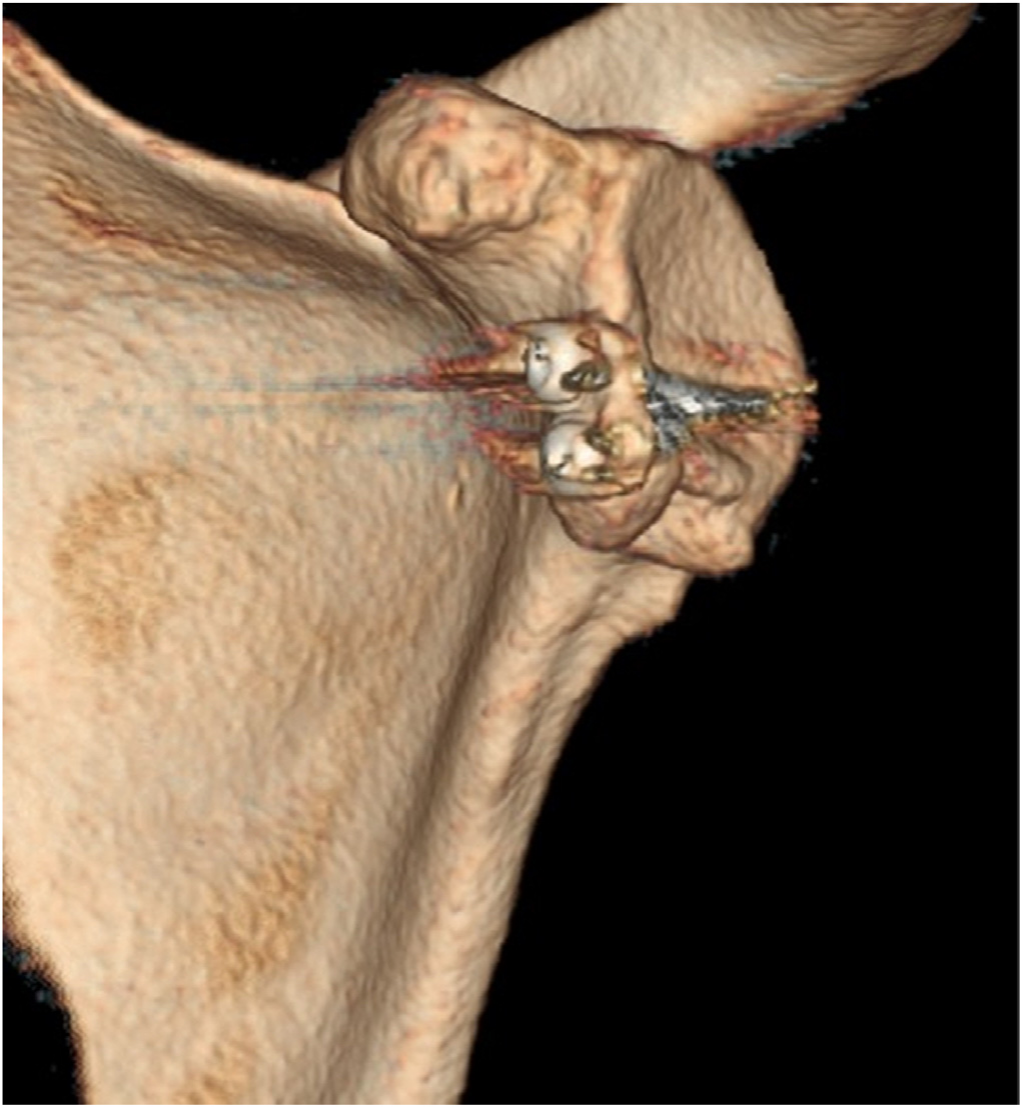
Three-dimensional CT scan image taken after posterior dislocation of anterior scapula and glenoid.

**Figure 9:**
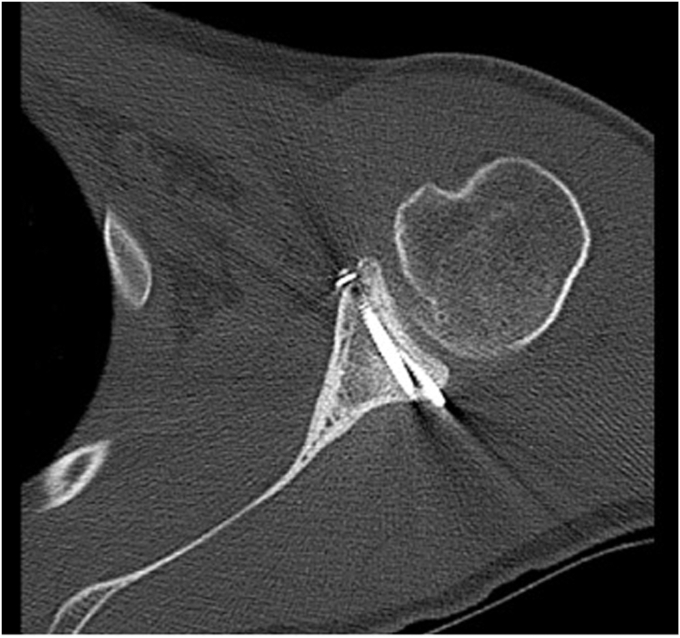
Axial CT image taken after posterior dislocation revealing reverse Hill-Sachs lesion.

Upon orthopedic evaluation, the patient’s shoulder demonstrated full ROM and good strength of the supraspinatus, infraspinatus, teres minor, subscapularis, as well as the anterior, middle, and posterior deltoid. However, he had evidence of posterior-inferior instability demonstrated by positive posterior apprehension, load-and-shift, and O’Brien’s tests. Three-dimensional computed tomography scan revealed a reverse Hill-Sachs lesion and bony deficiency of the posterior inferior glenoid (Figure 6, Figure 7, Figure 8, Figure 9). Treatment options were discussed, including an arthroscopic capsular shift with labral repair, but the patient desired to return to contact sports and weightlifting. Thus, the decision was made to undergo a posterior glenoid reconstruction with bone graft, capsular shift, and labral repair with possible removal of the Latarjet screws to make room for the posterior glenoid bony reconstruction.

Examination under anesthesia prior to surgery confirmed the shoulder was unstable in the posterior-inferior direction. The patient was placed in the lateral position and an inverted L incision was made just distal to the level of the spine of the scapula that extended towards the posterior acromion and from there, distally to the posterior axillary floor. Skin flaps were raised, and the posterior deltoid was retracted laterally so that the infraspinatus and teres minor were exposed. The interval between the teres minor and infraspinatus was developed such that the capsule was exposed. With elevation of the capsule, most of the posterior, inferior, and superior labrum appeared to be detached and there was an area of bony defect involving the posterior-inferior aspect of the glenoid measuring approximately 11 mm in length and 10 mm in width. This area was débrided, but it appeared that the screws from the anterior Latarjet procedure were prominent, and the decision was made to remove them. Débridement of the glenoid was performed at this time and the wound irrigated as attention was moved anteriorly for screw removal. The scar from his previous Latarjet was partially reopened and the deltopectoral interval developed. Deep dissection was performed until the level of the coracoid was reached. The inferior Latarjet screw was more posteriorly prominent and removed with its washer through an anterior approach. The proximal screw was well-seated in the bone and broke after partially disengaging the screw head from the bone such that most of the length of the screw remained inside the bone. Irrigation of the anterior wound was performed, and the rotator interval was closed with interrupted ultra-high-molecular-weight polyethylene suture in layered subcuticular fashion.

**Figure 10:**
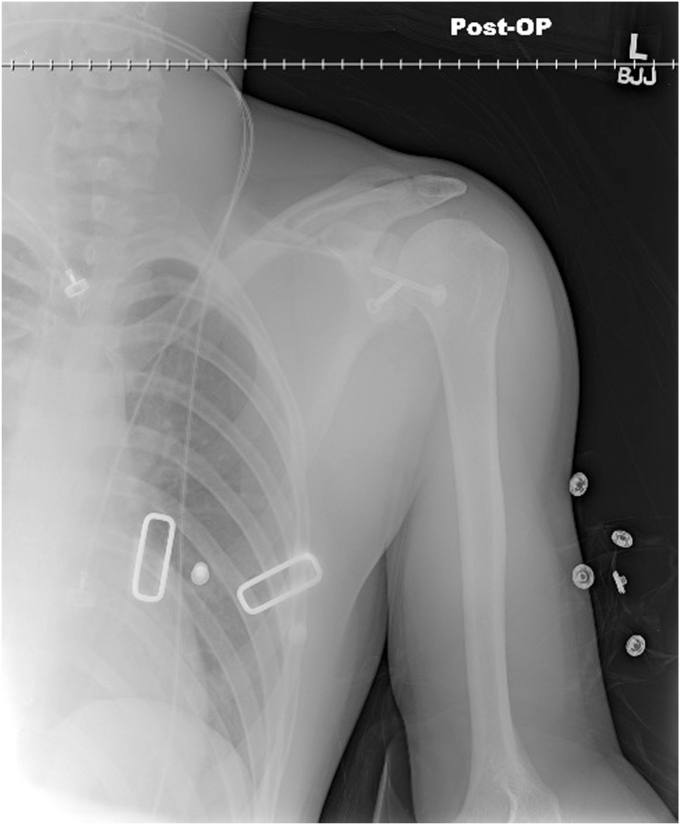
Anterior to posterior X-ray taken postoperatively after posterior glenoid reconstruction with scapular spine bone graft, capsular shift, and labral repair.

Attention was again directed posteriorly, and the capsule was opened longitudinally. The joint was exposed. The detached labrum was further separated to expose the undersurface, which was débrided with a burr. Three size 2.9 double-loaded suture anchors were placed with equal spacing superior, middle, and inferior to the posterior glenoid. The shoulder was then placed in 20 degrees of external rotation and the sutures from the anchor were used to repair the labrum and imbricate the redundant capsule. Attention was then directed toward bony reconstruction. Through the same exposure, the lower trapezius insertion over the medial spine of the scapula was elevated and approximately 15 mm by 12 mm of bone from the medial spine of the scapula was harvested to be used as a bone graft. After thorough irrigation of the site of the harvest, drill holes were placed medially, and the lower trapezius was reattached back to its anatomic location. The bone was contoured to fit the site of the bony defect and a cannulated 3.5 mm stainless steel screw and washer were placed (Fig. 10). Excellent compression was achieved at this level and the shoulder was stable to posterior and anterior stress testing. Prior to surgery, the shoulder easily subluxated posteriorly and inferiorly. Drill holes were placed in the spine of the scapula and used to repair the deltoid back to its anatomic location. Antibiotic powder was placed in the wound and the wound layers were closed in subcuticular fashion. Dressing and an external rotation brace was applied. The patient was awakened and transferred to recovery without complication.

Postoperative course was uneventful. After remaining in the external rotation brace for eight weeks, the patient quickly regained full ROM compared contralaterally (except for the internal rotation lost after the Latarjet) and gained approximately 5 degrees of external rotation compared contralaterally before beginning rotator cuff strengthening. By seven months postop, he had achieved preinjury strength. Upon last follow-up, which was approximately ten years after surgery, he has been able to do all desired physical activity without pain or instability. His American Shoulder and Elbow Surgeons score is 95, marking substantial improvement from his preoperative baseline of 75, his subjective shoulder value is 100, and his subjective shoulder value–Sport is 97.

## Discussion and conclusion

Treatment for recurrent shoulder instability is guided by a variety of factors. The ideal plan correctly pairs surgical technique with the underlying pathology in order to restore anatomical function. Determining the extent of soft tissue and bony involvement is crucial to creating a successful treatment plan, as these have widely differing contributions to instability due to their differing anatomical defects. Arthroscopic Bankart repairs are generally considered first line surgical management for recurrent anterior instability with soft tissue damage to the labrum and minimal (<20%) glenoid bone loss.^1^ Similarly, surgical management of tears in the glenohumeral ligament target the soft tissue defects within the joint. Soft tissue procedures are often preferred because complications are frequently less severe and more easily corrected than complications of bone augmentation procedures. However, the Bankart repair and similar soft tissue techniques are inferior to bone augmentation when there is significant bony involvement, as they do not address the bony anatomic defects. Though there is some debate as to the exact amount of glenoid bone loss that should be considered “critical” and how large on and off-track Hill-Sachs lesions must be to warrant fixation, there is universal agreement that they are important factors that contribute to recurrent anterior instability.^4^, ^5^, ^6^^,^^13^^,^^17^^,^^20^^,^^25^ Generally, 20-25% bone loss is considered enough to warrant bone augmentation, usually in the form of a bone graft or remplissage, with some surgeons even recommending a critical value as low as 17.3% (^3^^,^^17^^,^^26^^,^^27^). The Latarjet procedure is well-established as a first line surgical treatment for recurrent anterior shoulder instability with significant osseous defects and is often recommended to patients who are young and active, especially in contact sports since it has a substantially lower risk of recurrent instability compared to soft tissue procedures alone.^2^^,^^10^^,^^14^^,^^29^

Just as with recurrent anterior instability, the principles of treating recurrent posterior shoulder instability should focus on correcting the underlying pathology and restoring normal anatomy. However, this is often difficult as there is more ambiguity in treatment guidelines as well as a relative scarcity of posterior dislocations, and therefore surgeon experience.^8^^,^^18^ Arthroscopic reverse Bankart repairs, capsular plications, and repairs of the posterior band of the inferior glenohumeral ligament can be effectively utilized in patients with subcritical (generally regarded as <20%) glenoid bone loss with high success rates^9^ While associated with relatively high failure rates, posterior bone block procedures should be considered in the presence of critical (>20-25%) glenoid bone loss or large humeral head lesions and can utilize grafts from the iliac crest, distal tibia, scapular spine, or femur.^6^^,^^19^

After a thorough search, there is only one reported case of ipsilateral anterior and posterior shoulder dislocations. The reported patient was a 23-year-old male who was already set to have a posterior shoulder stabilization surgery when he was in a motor vehicle accident and suffered an ipsilateral anterior shoulder dislocation. To address both problems at the same time, an arthroscopically assisted Latarjet with posterior bone graft was performed. To the authors’ knowledge, that type of procedure had not been performed before.^7^ Our case is different as the time between the anterior glenoid and posterior glenoid augmentation was separated by several years and both were the result of separate traumatic injuries.

This case report shows that, when correctly utilized, anterior and posterior bony augmentation procedures can provide satisfactory outcomes, even allowing athletes to return to preinjury participation levels in high impact sports. Though often avoided for fear of complications and technical difficulty, this case demonstrates that correctly addressing underlying pathology with the most direct anatomical corrections can provide great outcomes and should be considered when managing recurrent anterior and posterior shoulder instability. Though Bankart and similar soft tissue techniques should be utilized when addressing primarily soft tissue injuries, bone augmentation must be considered when bone loss is apparent. There are no reported cases of an ipsilateral posterior dislocation after a Latarjet, so there is an obvious need for more patients to be studied before making conclusions regarding the long-term effectiveness of posterior bone grafting, especially when a Latarjet has already been performed. However, considering that our patient has had zero episodes of recurrent instability (defined as subluxations or dislocations) in the ten years that have elapsed since surgery and that most recurrent dislocations occur in the first two years after the initial dislocation, we believe that this case report can provide a helpful guide to surgeons managing anterior and posterior shoulder instability with bone loss.^11^

## Disclaimers:

Funding: The authors did not receive support from any organization for the submitted work.

Conflicts of interest: The authors, their immediate families, and any research foundation with which they are affiliated have not received any financial payments or other benefits from any commercial entity related to the subject of this article.

Patient consent: Obtained.
